# WT1 Inhibits Human Renal Carcinoma Cell Proliferation and Induces G2/M Arrest by Upregulating IL-24 Expression

**DOI:** 10.1155/2022/1093945

**Published:** 2022-07-23

**Authors:** Y. J. Jing, L. C. Lin, L. L. Chen, Z. E. Huang, H. C. Qin, S. B. Li, Z. H. Chen

**Affiliations:** ^1^Institute of Life Sciences, Youjiang Medical University for Nationalities, Baise, Guangxi, China; ^2^School of Basic Medicine, Youjiang Medical University for Nationalities, Baise, Guangxi, China

## Abstract

The transcription factor Wilms' tumor 1 (WT1) is involved in development, tissue homeostasis, and disease. However, the exact roles and the mechanisms of WT1 in renal carcinoma are not well understood. Therefore, in this study, we evaluated the ability of WT1 to block proliferation in renal carcinoma cells in vitro. Experimental analysis showed that WT1 overexpression inhibited the proliferation of renal carcinoma A498 cells and promoted arrest at the G2/M checkpoint. RNA-Seq identified differentially expressed genes, including IL-24, related to both the cell proliferation and the cell cycle. WT1 overexpression upregulated IL-24 expression, and IL-24 overexpression induced G2/M arrest. ChIP-Seq identified JUN as a direct target of WT1 in A498 cells, in which positive regulation was shown by RT-qPCR. It has been shown that the transcription factor JUN can regulate IL-24 expression, and therefore, we hypothesize that WT1 might regulate the IL-24 through JUN. Furthermore, analysis based on TCGA datasets showed that the expression of WT1-regulated genes, including TXNIP and GADD45A, was significantly correlated with the stage and histological grade of tumors, with high levels linked to favorable prognoses. Our results demonstrated that the overexpression of WT1 upregulates IL-24, leading to G2/M checkpoint arrest to reduce proliferation. These results indicate that regulation of IL-24 by WT1 inhibits proliferation and may represent a potential target for treating renal carcinoma.

## 1. Introduction

Renal cell carcinoma (RCC) is subdivided into clear cell RCC (KIRC), papillary RCC, and chromophobe RCC, with KIRC, accounting for 75-80% of cases, being the most frequent [1–3]. Although surgical resection may be effective, the tumor is often diagnosed late and frequently recurs, resulting in a poor prognosis. Therefore, it is urgent to elucidate the molecular mechanisms underlying tumorigenesis and progression of RCC to identify novel markers for both its diagnosis and its treatment. Excessive proliferation is one of the biological characteristics of malignant tumor cells.

Currently, many clinical anticancer drugs are designed to counteract proliferation, including paclitaxel, camptothecin, and vincristine. This indicates the usefulness of investigating the molecular mechanisms related to tumor proliferation for discovering new drug targets [4–6]. Wilms' tumor gene 1 (WT1) is a multifunctional transcriptional regulator involved in cell growth, differentiation, and apoptosis through the activation or inhibition of target genes. It is not only involved in the normal development of multiple organs but also closely related to the genesis and development of tumors [7, 8]. Initial research into the role of WT1 in tumors has focused on hematological-derived tumor cells. Many studies have shown that WT1 is highly expressed in human leukemia cells. In patients with chronic myelogenous leukemia (CML), the expression level of WT1 increased gradually from the chronic to the acute phase, parallel to the progression of the disease [9–11]. Subsequent studies have shown that in addition to leukemia cells, WT1 is overexpressed in a number of solid tumors, including tumors of the lung, breast, and colon, and can be used as a malignant tumor antigen for these tumors [12, 13]. Miyoshi et al. reported a relationship between high WT1 levels and unfavorable prognoses in breast cancer patients, with WT1 levels positively related to proliferation, while liposome encapsulation of a WT1 antisense deoxy-oligonucleotide was found to inhibit cell growth by downregulating WT1 expression [14]. These results suggest that WT1 contributes to cancer progression by stimulating cell proliferation. In contrast, some studies have shown that WT1 can inhibit tumor growth. For example, Wang and Wang have shown that WT1 preserves normal growth patterns in mammary epithelial cells, with downregulated expression associated with cancer development [15]. Therefore, whether WT1 acts as an inhibitor or promoter of tumor cell proliferation varies with different tumor tissues and is tissue-specific.

To date, the action of WT1 on RCC cells remains unknown. Based on the above research background, in this study, we investigated the role and mechanism of action of WT1 in RCC, showing that it acts as a potential tumor suppressor in this malignancy. We found that WT1 overexpression induces G2/M checkpoint arrest through the upregulation of IL-24, thereby inhibiting tumor cell proliferation. These findings contribute to the elucidation of the role of WT1 in RCC and suggest that targeting the WT1/IL-24 axis may be beneficial as an anticancer therapy.

## 2. Materials and Methods

### 2.1. Bioinformatic Analysis Methods

WT1 levels in different cancers were determined using the TIMER database (https://cistrome.shinyapps.io/timer/). Analysis of WT1 mRNA expression between normal tissues and KIRC cancer tissues was performed using the UALCAN database (http://ualcan.path.uab.edu/), which was used for mRNA expression between the KIRC and normal tissues, and the HPA database (https://www.proteinatlas.org/) was used for analyzing protein levels. The LinkedOmics database (http://www.linkedomics.org/login.php) was used to identify differentially expressed genes in relation to WT1 in TCGA KIRC cohort (*n* = 371). DAVID (http://david.abcc.ncifcrf.gov/) is a functional annotation tool for revealing the biological significance of genes. GO analysis includes three categories: biological processes (BP), cellular components (CC), and molecular functions (MF). The KEGG database was used to identify biological pathways for gene enrichment. Fisher's exact test was used to measure significant differences, and *p* values < 0.05 were considered significant.

### 2.2. Cell Culture, Plasmid Construction, and Transfection

The human renal cell carcinoma cell line A498 was purchased from Wuhan Pu-nuo-sai Life Technology Co. Ltd. (Wuhan, China) and maintained in the minimum essential medium (MEM) supplemented with 10% fetal bovine serum (FBS) and 100 U/ml of penicillin-streptomycin. The cells were incubated at 37°C in a humidified atmosphere with 5% CO_2_. In addition, the cell line was not contaminated or misidentified according to the Database of Cross-Contaminated or Misidentified Cell Lines. The pReceiver-M93-WT1, pReceiver-M93-IL-24, and empty vector plasmids were purchased from GeneCopoeia, Inc. (Guangzhou, China) and transfected as described previously [16].

### 2.3. ELISA

ELISA kits for IL-6 assay were purchased from Neobioscience Co., Ltd. (Shenzhen, China). Culture supernatants were added to 96-well plates and incubated with an anti-IL-24 antibody for 30 min at 37°C. The plates were incubated with the secondary HRP-conjugated antibody under the same conditions. After incubation with TMB for 20 min at room temperature away from light, the reaction was stopped, and the absorbance at 450 nm was read. The IL-24 concentrations were determined from a standard curve.

### 2.4. Western Blotting Analysis

Protein was extracted using the RIPA lysis buffer, and protein concentrations were measured using the BCA method. Proteins were separated on 10% SDS-PAGE and blotted onto PVDF membranes. Anti-Tublin (Abcam, Cambridge, UK) and anti-Flag (Abcam) antibodies were used to probe the blots. The blots were incubated with the appropriate secondary antibodies and visualized by chemiluminescence.

### 2.5. RNA-Seq and Data Analyses

Total RNA was extracted using TRIzol (Life Technologies Corp.), and genomic DNA was removed by DNase treatment. mRNA was isolated using the NEBNext Poly(A) mRNA Magnetic Isolation Module (New England Biolabs, Ipswich, MA, USA) and RNA-Seq libraries prepared using the NEBNext Ultra Directional RNA Library Prep Kit for Illumina (New England Biolabs). Illumina sequencing was performed using paired-end 2 × 150 as the sequencing mode. High-quality reads were prepared by removal of sequencing adapters with short reads (<35 bp), while low-quality reads using Cutadapt (v1.9.1) and Trimmomatic (v0.35), and the quality was assured with FastQC. The clean reads were mapped to the mouse genome (assembly GRCm38) with HISAT2 software. The expression levels of genes were determined using FPKM (fragments per kilobase of exon per million fragments mapped) by StringTie. Differential expression was assessed by Ballgown in R. The false discovery rate (FDR) method was used to calculate the adjusted *p* values in multiple testing to determine the significance of the differences, and only genes with adjusted *p* values < 0.05 were used for subsequent analysis.

### 2.6. Measurement of Proliferation and Cell Cycle Analysis

Bioluminescence imaging was used to measure proliferation. The A498-Luc2 cells were inoculated in 96-well plates and transfected with either the WT1 or the control vector. D-Luciferin (150 mg/ml) was added to the wells, and the photons were counted after 5 min in an IVIS Lumina LT system (PerkinElmer) with data analysis by Living Image software. Cell cycle analysis was performed as described previously [16].

### 2.7. ChIP-Seq Library Preparation

Cells were crosslinked with 1% formaldehyde for 10 min at room temperature and quenched with 125 mM glycine. The fragmented chromatin fragments were precleared and then immunoprecipitated with Protein A+G Magnetic Beads coupled with anti-Flag antibodies. After reverse crosslinking, ChIP and input DNA fragments were end-repaired and A-tailed using the NEBNext End Repair/dA-Tailing Module (E7442, NEB), followed by adapter ligation with the NEBNext Ultra Ligation Module (E7445, NEB). The DNA libraries were amplified for 15 cycles and sequenced using Illumina NovaSeq PE150 as the sequencing mode.

### 2.8. ChIP-Seq Analysis

ChIP DNA samples were subjected to end repair and A-base addition, followed by ligation with adapters. We defined target genes as those that contain ChIP-Seq peaks located within transcribed regions of genes, in introns or 3 kb upstream of the TSS or 3 kb downstream of the TTS.

### 2.9. Mapping of Paired-End Reads

Before read mapping, clean reads were obtained from the raw reads by removing the adapter sequences. The clean reads were then aligned to reference genome sequences using the BWA software. Peak detection was performed using the MACS2 software peak finding algorithm with 0.01 set as the *p* value cutoff.

### 2.10. RNA Isolation and Quantitative Real-Time- (RT-) PCR (RT-qPCR)

An RNeasy Mini Kit (Qiagen, Valencia, CA, USA) was used to extract total cellular RNA using the supplied protocol. The All-in-One First-Strand cDNA Synthesis Kit (GeneCopoeia, Guangzhou, China) was then used to reverse-transcribe the cDNA. The primers were purchased from GeneCopoeia Co., Ltd. (Guangzhou, China): human WT1 (HQP058379), human IL-24 (HQP063970), human JUN (HQP009854), human JUND (HQP062670), human TXNIP (HQP090833), human GADD45A (HQP055169), and human GAPDH (HQP064347). The All-in-One qPCR Mix Kit (GeneCopoeia) was used for RT-qPCR using a Roche LightCycler 96 (Roche).

### 2.11. Statistical Analysis

Data are presented as the mean ± standard deviation (SD) and were analyzed using GraphPad Prism 6.0. Differences were determined by one-way ANOVA with *p* < 0.05 considered significant.

## 3. Results

### 3.1. Downregulation of WT1 Expression in KIRC

**Figure 1:**
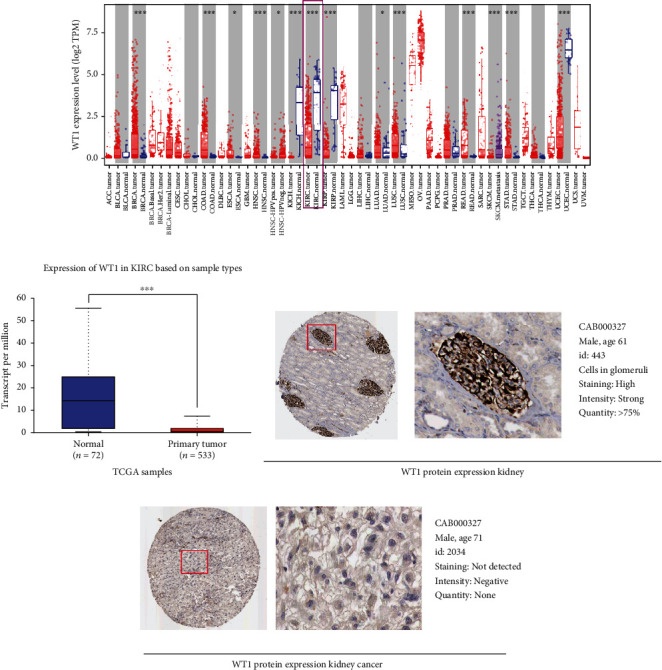
WT1 levels in normal and KIRC tissues. (a) WT1 levels in various cancers from the TIMER database. (b) WT1 levels in renal cancer from the UALCAN database. (c) WT1 protein levels in renal cancer from the HPA database.

We first analyzed the expression profile of WT1 using the TIMER (Tumor Immune Estimation Resource) database. WT1 levels were significantly reduced in several solid tumors, including KIRC, KIRP, KICH, and UCEC tissues (Figure 1(a)). The WT1 levels in RCC tissues were investigated using the UALCAN (http://ualcan.path.uab.edu) database showing that WT1 mRNA was significantly raised in KIRC in comparison with normal tissues (Figure 1(b)). We then used the Human Protein Atlas database to examine WT1 protein levels. WT1 protein was not expressed in KIRC, while high levels were seen in the normal kidney (Figure 1(c)). These results indicate that WT1 is poorly expressed in KIRC, suggesting that its downregulation may be important for RCC progression.

### 3.2. Functional Association of WT1-Related Genes with Cell Proliferation in KIRC

**Figure 2:**
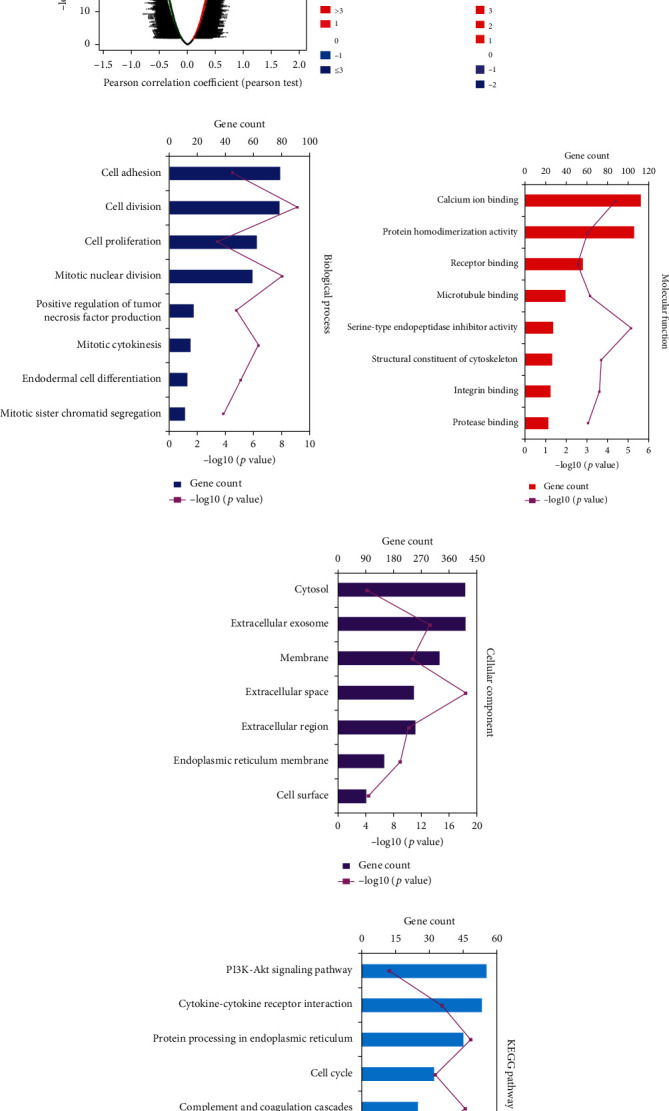
KEGG and GO enrichment analyses of WT1-related genes showing their relationship with cell proliferation. (a) Pearson correlations between WT1 and differentially expressed genes. (b) Heatmap of the top 50 positively correlated genes. (c–f) DAVID analysis of significantly enriched GO annotations and KEGG pathways. (c) Biological processes, (d) molecular functions, (e) cellular components, and (f) KEGG pathway analysis.

The function module of LinkedOmics was used to analyze mRNA sequencing data from 533 KIRC patients in TCGA. The volcano plot (Figure 2(a)) illustrates the correlations between genes and WT1: 5855 genes (dark-red dots) were positively correlated while 3470 (dark-green dots) were negatively correlated (false discovery rate). The heatmap (Figure 2(b)) illustrates 50 gene sets positively and negatively correlated with WT1. These findings suggest that WT1 has an extensive influence on the transcriptome. Gene ontology (GO) analysis found that differentially expressed genes that were positively related to WT1 were largely located in the cytosol, extracellular exosome, and extracellular region, where they are primarily involved in cell adhesion, cell proliferation, and mitotic nuclear division (Figures 2(c)–2(e)). KEGG pathway analysis demonstrated enrichment in the p53 signaling pathway and cell cycle pathways (Figure 2(f)).

### 3.3. WT1 Overexpression Can Inhibit RCC Cell Proliferation by Inducing G2/M Arrest

**Figure 3:**
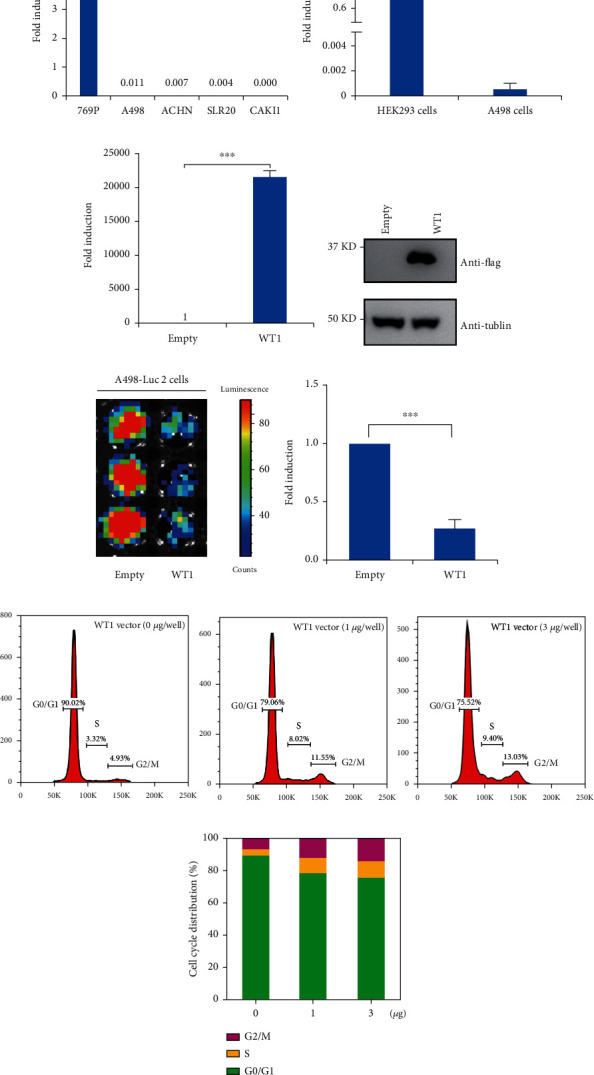
WT1 overexpression inhibits the proliferation of A498 cells by promoting G2/M arrest. WT1 levels in renal cancer cell lines from the CCLE database (a). WT1 mRNA levels measured by RT-qPCR (b). Cells were transfected with WT1 or control vectors (2 *μ*g/well), and WT1 levels were measured by RT-qPCR (c) or Western blotting (d). A498-Luc2 cells transfected with the WT1 vector or empty vector (0.2 *μ*g/well). FLUX measurements were captured (e). For normalization of luciferase activity, the luciferase signal per well of the control cells was set to 1. Quantitative data represent the mean ± standard error (*n* = 3 per group) (f). Cell cycle analysis of WT1-overexpressing A498 cells by flow cytometry. Flow cytometry of WT1-overexpressing A498 cells showing G2/M arrest was assessed via flow cytometry. Histograms of cell cycle distribution (g). Statistical diagram of the cell cycle distribution (h). Experiments were conducted in triplicate (*n* = 3); error bars represent standard errors; ^∗∗∗^*p* < 0.001.

Next, we asked whether WT1 overexpression can inhibit the proliferation of human RCC cells. We first analyzed WT1 mRNA levels in several RCC cell lines using the CCLE database, which showed low WT1 expression in A498 cells (Figure 3(a)). We then used RT-qPCR to analyze WT1 expression in A498 cells and HEK293 cells (normal embryonic kidney cells), observing significantly lower levels of WT1 in A498 cells, consistent with the CCLE database analysis (Figure 3(b)). To determine the inhibitory effect of WT1 on RCC cells, we constructed a WT1-overexpressing vector (pReceiver-M93-WT1). This vector was transfected into A498 cells, and the effect was validated by RT-qPCR or Western blotting (Figures 3(c) and 3(d)). Next, we used bioluminescence imaging to assess the effect of WT1 overexpression on proliferation. Luciferase-expressing A498 cells were transiently transfected, and alterations in signal intensity, as a measure of the cell number, were examined. Approximately fourfold diminished signals were observed in the WT1-overexpressing cells in comparison with cells transfected with an empty vector after 48 h (Figures 3(e) and 3(f)), demonstrating that WT1 overexpression reduces RCC cell proliferation. Flow cytometry showed that WT1 overexpression resulted in increased numbers of cells at the G2/M checkpoint in comparison with controls (Figures 3(g) and 3(h)). Taken together, these data suggested that WT1 overexpression reduces RCC cell proliferation by G2/M arrest.

### 3.4. Transcriptomic Analysis of WT1-Regulated Genes in RCC Cells

**Figure 4:**
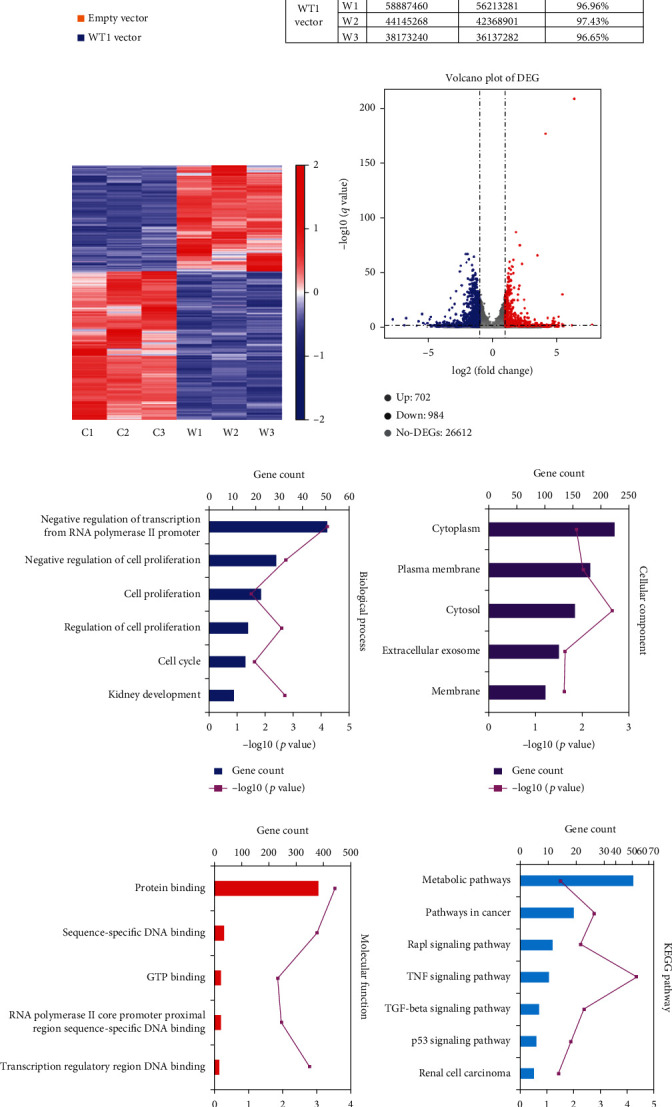
Transcriptomic analysis of WT1-regulated genes. (a) The mRNA expression level of WT1 in A498 cells after transfection with the WT1 vector or empty vector after 48 h was detected by RT-qPCR. (b) Data and flowchart of AmpliSeq arrays assessing gene expression in triplicate biological replicates (W1-W3: WT1 vector; C1-C3: empty vector) in A498 cells. (c) Volcano plot of the transcriptome. (d) Volcano plot of the transcriptome. Upregulated (blue color) and downregulated (red color) genes in A498 cells transfected with WT1 along with statistically nonsignificant expressed genes (gray color) are plotted. (e–h) Significantly enriched GO annotations and KEGG pathways of WT1-related genes analyzed by DAVID. (e) Biological processes, (f) molecular functions, (g) cellular components, and (h) KEGG pathway analysis. ^∗∗∗^*p* < 0.001.

The total RNA isolated from A498 cells transfected with the empty vector or the pReceiver-M93/Flag-WT1 vector was analyzed using RNA-Seq.  Figure 4(a) illustrates the increased WT1 levels after transfection with the WT1 vector compared with controls. Targeted enrichment of 21,000 genes produced 10.5 to 37.8 M reads, with approximately 97% aligning to the human reference genome (Figure 4(b)). WT1 levels in overexpressing cells (W1, W2, and W3) were normalized with controls (C1, C2, and C3) and led to the identification of 702 upregulated (blue dots) and 984 downregulated (red dots) genes, with gray dots indicating genes that remained unchanged. These results suggest that WT1 may regulate transcription both positively and negatively, as previously described [17] (Figures 4(c) and 4(d)). GO analysis of the high-confidence WT1 targets showed that these genes were located mainly in the cytosol, cytoplasm, and plasma membrane, where they participated primarily in the “cell cycle,” “negative regulation of cell proliferation,” and “negative regulation of transcription from RNA polymerase II promoter” (Figures 4(e)–4(g)). KEGG pathway analysis showed enrichment in the “renal cell carcinoma,” “metabolic pathways,” “p53 signaling pathway,” and “TGF-*β* signaling pathway” (Figure 4(h)).

### 3.5. WT1 Overexpression Can Induce G2/M Arrest by Upregulating IL-24 Expression in RCC Cells

**Figure 5:**
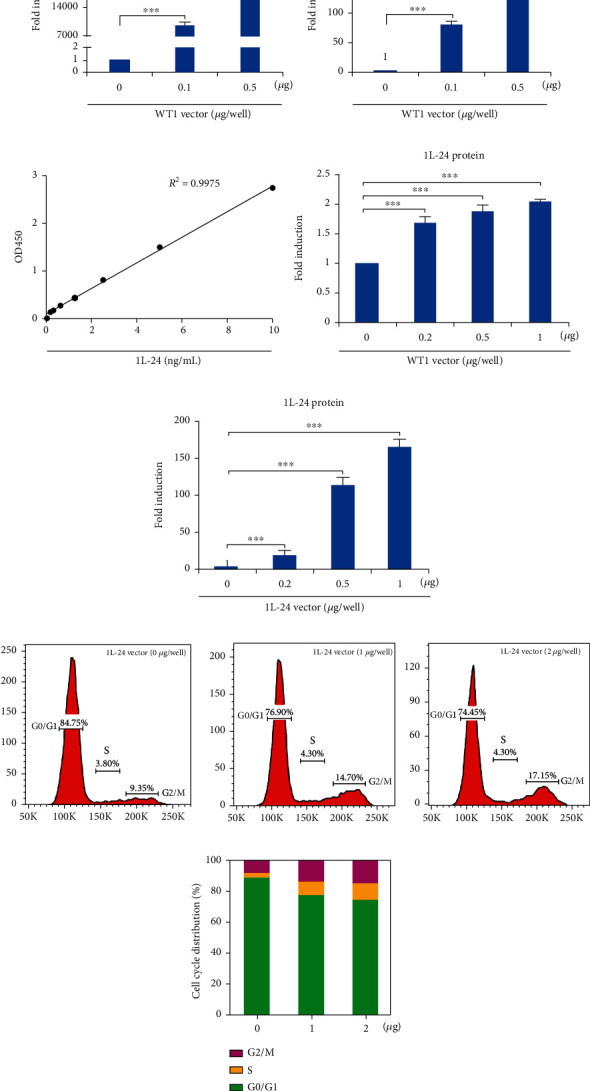
WT1 overexpression can induce G2/M arrest by upregulating IL-24 expression in RCC cells. The expression of (a) WT1 or (b) IL-24 mRNA in A498 cells transfected with the WT1 vector was detected by RT-qPCR. (c) Standard curve for ELISA-based detection of IL-24 levels. (d) The expression of IL-24 protein in A498 cells transfected with the WT1 vector was detected by ELISA. (e) IL-24 levels in IL-24-overexpressing cells detected by ELISA. Flow cytometry of IL-24-overexpressing A498 cells showing G2/M arrest was assessed via flow cytometry. (f) Histograms of cell cycle distribution. (g) Statistical diagram of the cell cycle distribution. Experiments were conducted in triplicate (*n* = 3); error bars represent standard errors; ^∗∗∗^*p* < 0.001.

IL-24 is documented to inhibit growth and promote apoptosis in cancer cells [18–20]. In the current study, the RNA-Seq analysis revealed that WT1 overexpression induced the significant upregulation of IL-24 mRNA expression in A498 cells, and this result was confirmed by RT-qPCR (Figures 5(a) and 5(b)). Since IL-24 is a secreted protein, the IL-24 level was detected by ELISA in this study. This result showed that IL-24 levels were significantly increased in relation to WT1 concentration (Figures 5(c) and 5(d)). Next, we asked whether IL-24 overexpression can induce G2/M arrest in RCC cells. To answer that question, A498 cells were transfected with an IL-24 vector, and IL-24 levels were measured by ELISA. It was observed that the IL-24 protein level was significantly increased compared relative to the control cells (Figure 5(e)). Flow cytometry showed that IL-24 overexpression led to increased numbers of cells at the G2/M checkpoint (Figures 5(f) and 5(g)). These findings suggest that the overexpression of IL-24 promotes G2/M arrest in RCC cells.

### 3.6. Genome-Wide Identification of WT1 DNA Binding in RCC Cells

**Figure 6:**
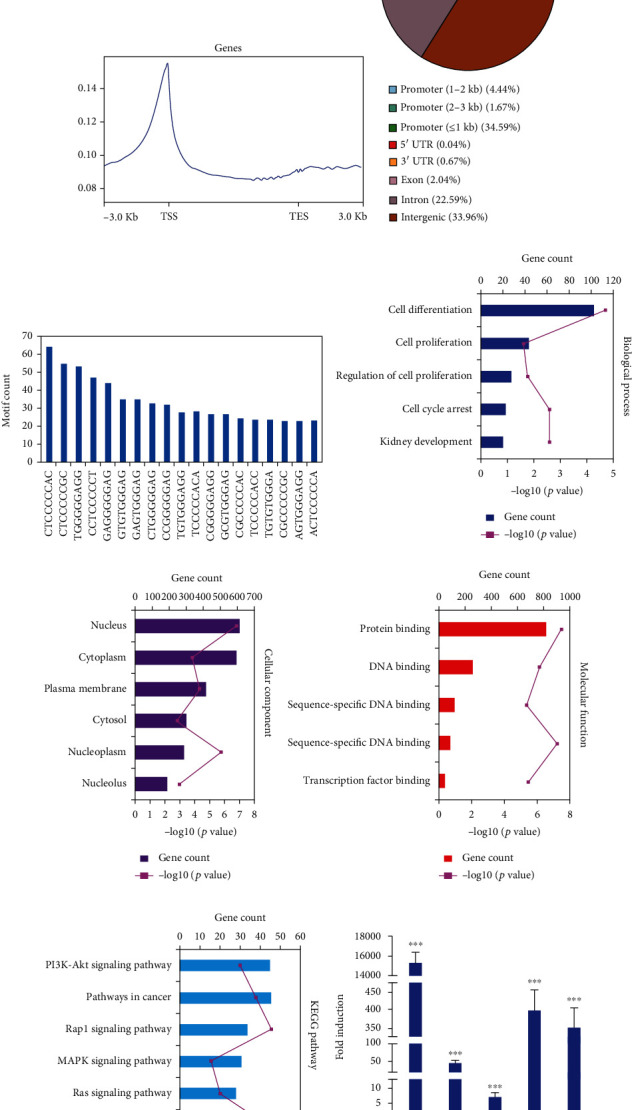
Genome-wide WT1 association in renal cancer cells. (a) Table showing data and flow of analysis of the ChIP-Seq experiment from sequence reads to annotated gene assignment of the WT1-binding sites. (b) Analysis of the motifs associated with WT1 peaks showed enrichment for known WT1-binding sequences. (c) Distribution of WT1-binding regions in the renal cancer cell genome. (d) Distribution of WT1 promoter peaks relative to the TSS. (e) WT1-binding motifs identified by ChIP-Seq analysis. (f–i) Significantly enriched GO annotations and KEGG pathways of WT1-related genes analyzed by DAVID. (f) Biological processes, (g) molecular functions, (h) cellular components, and (i) KEGG pathway analysis. (j) The expression level of WT1-target genes was verified in WT1-overexpressing cells by RT-qPCR. Experiments were conducted in triplicate (*n* = 3); error bars represent standard errors; ^∗∗∗^*p* < 0.001.

To investigate the WT1-regulated genes involved in inhibiting RCC cell proliferation, we performed ChIP-Seq analysis. This showed close to 2700 sequence peaks, with a steady alignment rate of the reads including approximately 90% of DNA samples. Motif analysis was used to identify enrichment of the WT1-binding sequence (Figures 6(a) and 6(b)). Sixty-six percent of these sites were located in the genic regions of 1143 genes, with genic regions defined as the gene itself together with 3 kb upstream and downstream (Figure 6(c)). Among these, 40.71% of sites were situated in promoter regions (≤3 kb of the transcription start site), 0.04% in 5′-untranslated regions, 22.59% in intronic regions, 2.04% in exons, and 0.67% in 3′-untranslated regions, and 33.96% were intergenic (Figure 6(d)). The results suggested that WT1-binding sites were significantly enriched in the promoter regions. It has been reported that WT1 recognizes and binds to the DNA sequence 5′-GCGGGGGCG-3′ to regulate the expression of numerous target genes [21]. In the present study, the motif analysis showed that WT1 and its corresponding 590 motif sequences could be identified, including 5′-GCGGGGGCG-3′ sequences, which had been previously reported (Figure 6(e)). Next, we performed GO analysis of the WT1 targets which showed that WT1 target genes were associated with “cell differentiation,” “cell cycle arrest,” and “cell proliferation” (Figures 6(f)–6(h)). KEGG pathway analysis showed enrichment in the “pathways in cancer,” “p53 signaling pathway,” and “TGF-*β* signaling pathway” (Figure 6(i)). The ChIP-Seq results confirmed that WT1-regulated target gene functions were closely related to “cell proliferation” and “cell cycle arrest.” WT1 target genes, including JUN and JUND, were identified, and further RT-qPCR results confirmed that WT1 overexpression could upregulate the expression of these genes (Figure 6(j)).

## 4. Discussion

Transcription factors modulate the transcription of target genes and may thus play significant roles in tumor development [22]. WT1 is a zinc finger transcription factor that acts as both a tumor suppressor and an oncogene, depending on different cancers, so it is also called a chameleon gene [23]. Although previous studies have reported the inhibitory effect of WT1 in Wilms' tumor, its role in renal cancer remains unclear. In the current era of big data, bioinformatic analysis is critical to explore the molecular mechanisms of disease. In the present study, to gain insight into the actions of WT1 in RCC, we performed bioinformatic analysis of data from TCGA to show that while strongly expressed in most solid tumors, including BRCA, COAD, ESCA, HNSC, LUAD, LUSC, READ, and STAD, expression was low in renal cancers, including KICH, KIRC, KIRP, and UCEC tissues. GO analysis showed that WT1 was closely associated with cell proliferation-related pathways, including cell proliferation, cell division, and mitotic nuclear division; KEGG analysis indicated that WT1 was involved in the p53 signaling pathway, cell cycle pathway, and PI3K-Akt signaling pathway in KIRC. Further, flow cytometric analyses, Western blotting, and bioluminescence imaging confirmed that WT1 overexpression reduces proliferation in RCC cells by promoting G2/M checkpoint arrest. The results suggest that WT1 acts as a tumor suppressor in human RCC cells.

Transcriptome sequencing using RNA-Seq is generally considered the most effective method for mining functional genes [24]. Here, we comprehensively analyzed the WT1 regulatory profile using RNA-Seq, resulting in the identification of 1686 differentially expressed genes between cells transfected with the WT1 vector versus the empty vector. Previous studies have shown that WT1 can promote cancer progression by regulating target genes, such as p21, SLUG, and CDH1 [8]. However, our RNA-Seq data did not identify significant alterations in these genes in A498 cells. This may be because of different regulatory networks in different cells. Even for the same transcription factor, the target gene regulated by it may be different from cell to cell. Thus, whether WT1 acts as an oncogene or tumor suppressor in tumor cells depends on the regulatory network of genes in different cells, which may be an important reason why WT1 is known as the chameleon gene. Conversely, RNA-Seq data showed that WT1 overexpression leads to changes in the expression of many cell cycle-related genes including IL-24. IL-24 is a unique tumor-suppressing cytokine, which can inhibit tumor growth, invasion, metastasis, and angiogenesis and induce apoptosis through autocrine-paracrine regulation. It can selectively inhibit growth in a variety of tumor cells without harming normal cells and is, therefore, an excellent target for anticancer therapy [25]. IL-24 shows low expression in many tumor cells, and the mechanism regulating its expression is still unclear. Here, we unexpectedly found that WT1 overexpression could significantly upregulate IL-24 in RCC by RNA-Seq analysis, and the result was confirmed by RT-qPCR and ELISA. Several studies have shown that IL-24 can selectively block proliferation by promoting G2/M arrest in many tumor cells, such as breast, lung, and liver cancer cells [20, 26, 27]. To investigate whether WT1's actions on proliferation are mediated by the upregulation of IL-24, we used flow cytometry to assess the effects of IL-24 in RCC cells, showing that its overexpression induced G2/M arrest and thus that a WT1/IL-24/G2/M-phase arrest regulatory axis exists in RCC cells.

To further elucidate the molecular mechanism of WT1 upregulating IL-24 expression, we performed WT1 ChIP-Seq with an anti-Flag antibody in A498 cells. DNA-binding motif analysis revealed numerous WT1-binding DNA sequences. WT1 recognizes *cis*-elements through the previously identified WT1-binding motif CGCCCCCGC, as well as others, including CTCCCCCA(G)C, GTGTGGGAG, GAGTGGGAG, and TGTGGGAGG. Among these, CTCCCCCA(G)C had the largest number of interactions, higher than CGCCCCCGC, and may, therefore, be a stronger binding site than CGCCCCCGC. The CT(G)CCCCCA(G)C motif appears to be the most conserved. The ChIP-Seq analysis confirmed many WT1 target genes identified in previous studies, for example, JUN, SMAD6, and TGFB [8]. We identified several additional target genes including JUND, but not IL-24, suggesting that IL-24 may not be directly regulated by WT1. The predicted results from the ChEA3 databases showed that JUN might be an important transcription factor for IL-24, and evidence suggests that the JUN transcription factor could directly regulate the expression of IL-24 by binding to the gene promoter [28]. Thus, we hypothesized that WT1 may indirectly regulate the expression of IL-24 by regulating JUN.

In addition to IL-24, RNA-Seq and RT-qPCR verification showed that WT1 overexpression could significantly upregulate TXNIP and GADD45A expression in A498 cells. It is well known that GADD45A and TXNIP are tumor suppressor genes. GADD45A acts as a proapoptotic protein that induces apoptosis and cell cycle arrest, and TXNIP is an effector in glucose metabolism and can thus inhibit tumor cell proliferation through glucose metabolism [29, 30]. The predicted results from the ChEA3 databases showed that JUN or JUND might be the transcription factor for TXNIP or GADD45A, and thus, we hypothesized that WT1 may indirectly regulate the expression of TXNIP or GADD45A by regulating JUN or JUND and thus inhibit the proliferation of RCC cells. Since WT1 and IL-24 are rarely expressed in KIRC tissues, we investigated the clinical prognostic significance of WT1-regulated genes, including GDD45A and TXNIP, finding that the levels of these genes were associated with the histological grade, tumor stage, and metastasis in KIRC patients. Furthermore, the survival of patients with low GDD45A or TXNIP levels was significantly worse (Supplementary Figure S1). These results suggest that WT1-regulated genes, such as TXNIP and GADD45A, could be used as prognostic biomarkers in renal cancer.

In summary, we demonstrated for the first time that WT1 overexpression can induce G2/M-phase cell cycle arrest through the upregulation of IL-24 to inhibit the proliferation of RCC cells. These results enhance our understanding of the mechanisms by which WT1 inhibits tumor cell proliferation, and this may provide useful ideas for the development of antitumor drugs. It is well known that IL-24 is an inflammatory cytokine closely involved in renal cancer progression. The conclusion of a WT1/IL-24 regulatory axis indicates the importance of WT1 in regulating the renal cancer microenvironment. Furthermore, our results also showed that WT1 was closely related to glucose metabolism pathways in renal cancer cells, suggesting that in addition to its effects on the tumor microenvironment, WT1 is also involved in the progression of renal cancer by regulating glucose metabolism pathways, which needs to be confirmed by further studies.

## 5. Conclusions

This study provides the first evidence that WT1 overexpression induces G2/M cell cycle arrest via upregulating IL-24 expression and inhibits human renal carcinoma cell proliferation. The findings suggest that the WT1/IL-24 regulatory axis may be essential as a regulator of cell proliferation and may thus represent an attractive target for renal carcinoma prevention and treatment.

## Data Availability

The data used to support the findings of this study are available from the corresponding author upon request.

## Supplementary Materials

Supplementary Materials Supplementary Figure S1: the relationships between WT1-related gene expression and clinical characteristics and their prognostic value in KIRC patients.
