# Supplementary material for: WT1 Inhibits Human Renal Carcinoma Cell Proliferation and Induces G2/M Arrest by Upregulating IL-24 Expression
Source: Biomed Res Int. 2022 Jul 23;2022:1093945. doi: 10.1155/2022/1093945 (PMC9338855; doi:10.1155/2022/1093945)
Supplement: Supplementary Materials — Supplementary Figure S1: the relationships between WT1-related gene expression and clinical characteristics and their prognostic value in KIRC patients. [file 1093945.f1.docx]

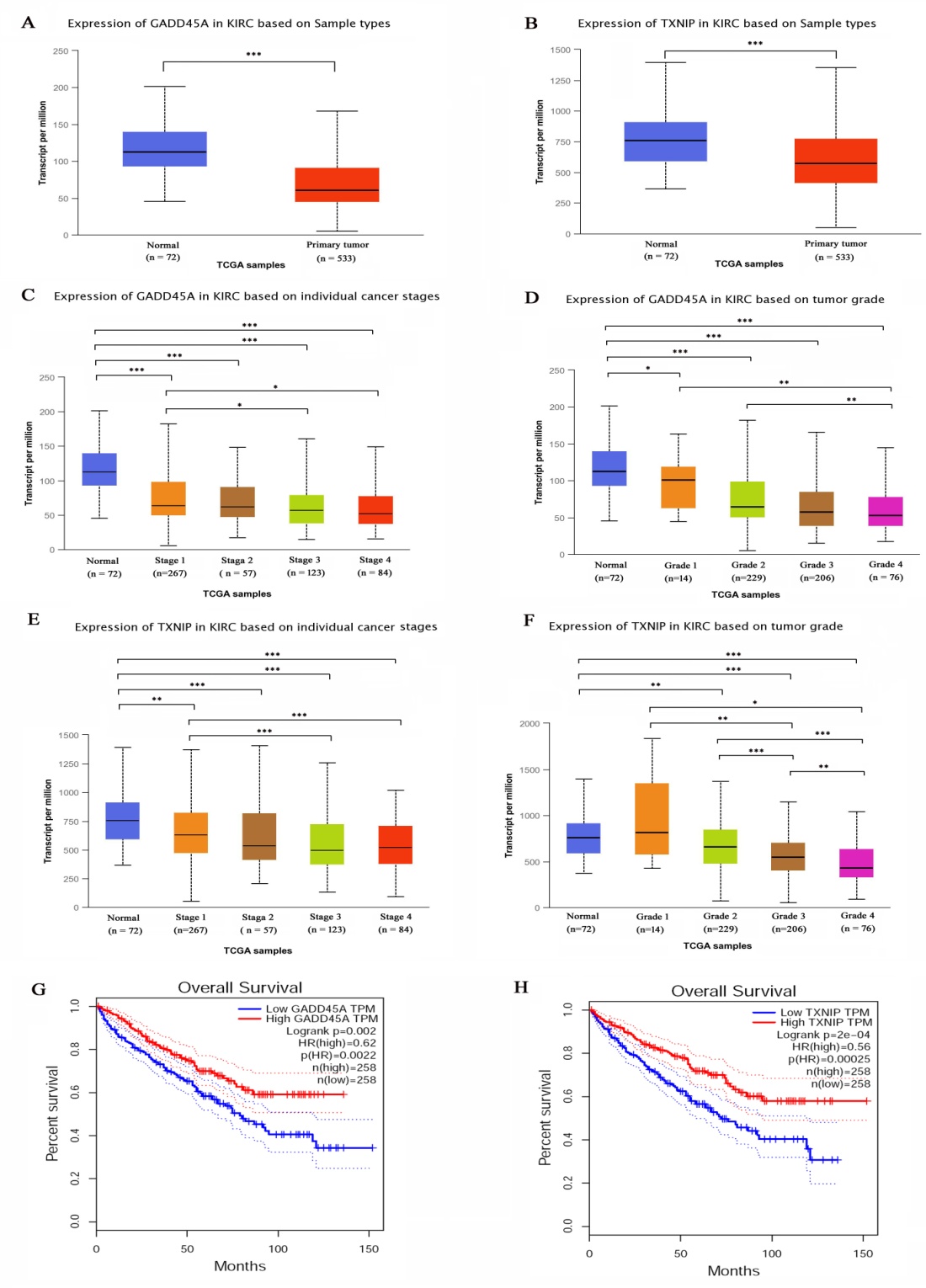


**Figure S1** The relationships between WT1-related gene expression and clinical characteristics and their prognostic value in KIRC patients. (A, B) Boxplot showing mRNA levels of GADD45A (A) or TXNIP (B) in KIRC tissues or adjacent tissues, from the UALCAN database. (C, D) Boxplot showing relative expression of GADD45A in normal individuals or KIRC patients with grade (C) or stage (D) tumors, from the UALCAN database. (E, F) Boxplot showing relative expression of TXNIP in normal individuals or KIRC patients with grade (E) or stage (F) tumors, from the UALCAN database. (G, H) Survival of patients with significant difference of TXNIP (G) or GADD45A (H) expression between KIRC tissues and adjacent tissues, from the GEPIA database. *, p < 0.05; **, p < 0.01; ***, p < 0.001.
